# Flaws in current human training protocols for spontaneous Brain-Computer Interfaces: lessons learned from instructional design

**DOI:** 10.3389/fnhum.2013.00568

**Published:** 2013-09-17

**Authors:** Fabien Lotte, Florian Larrue, Christian Mühl

**Affiliations:** Inria Bordeaux Sud-Ouest/LaBRITalence, France

**Keywords:** Brain-Computer Interface, instructional design, electroencephalography, training protocols, feedback

## Abstract

While recent research on Brain-Computer Interfaces (BCI) has highlighted their potential for many applications, they remain barely used outside laboratories. The main reason is their lack of robustness. Indeed, with current BCI, mental state recognition is usually slow and often incorrect. Spontaneous BCI (i.e., mental imagery-based BCI) often rely on mutual learning efforts by the user and the machine, with BCI users learning to produce stable ElectroEncephaloGraphy (EEG) patterns (spontaneous BCI control being widely acknowledged as a skill) while the computer learns to automatically recognize these EEG patterns, using signal processing. Most research so far was focused on signal processing, mostly neglecting the human in the loop. However, how well the user masters the BCI skill is also a key element explaining BCI robustness. Indeed, if the user is not able to produce stable and distinct EEG patterns, then no signal processing algorithm would be able to recognize them. Unfortunately, despite the importance of BCI training protocols, they have been scarcely studied so far, and used mostly unchanged for years. In this paper, we advocate that current human training approaches for spontaneous BCI are most likely inappropriate. We notably study instructional design literature in order to identify the key requirements and guidelines for a successful training procedure that promotes a good and efficient skill learning. This literature study highlights that current spontaneous BCI user training procedures satisfy very few of these requirements and hence are likely to be suboptimal. We therefore identify the flaws in BCI training protocols according to instructional design principles, at several levels: in the instructions provided to the user, in the tasks he/she has to perform, and in the feedback provided. For each level, we propose new research directions that are theoretically expected to address some of these flaws and to help users learn the BCI skill more efficiently.

## 1. Introduction

Brain-Computer Interfaces (BCI) are communication systems that enable users to send commands to a computer by using only their brain activity, this activity being generally measured using ElectroEncephaloGraphy (EEG) [see McFarland and Wolpaw (2011) for a review]. BCI have been shown to be very promising, notably for communication and control applications for severely disabled users (Wolpaw et al., 2002), but also in numerous other applications, such as rehabilitation (Pfurtscheller et al., 2008), human-computer interaction (Tan and Nijholt, 2010) or entertainment (Lécuyer et al., 2008), among many other (van Erp et al., 2012). Despite this potential, most BCI applications remain prototypes that are not used in practice, outside laboratories. The main reason is the widely acknowledged low reliability and low robustness of current BCI systems, especially as compared to alternative interfaces, e.g., computer mice or eye trackers. Indeed, the brain activity patterns produced by the user (e.g., resulting from imagining left hand movement to move a cursor toward the left) are too often incorrectly recognized by the BCI (McFarland and Wolpaw, 2011). These poor performances are due in part to the imperfect signal processing algorithms used to analyze and classify EEG signals. Indeed, these algorithms are not yet able to extract robustly the relevant information from EEG signals in the presence of various noise sources, signal non-stationarity and with limited amount of data available (McFarland and Wolpaw, 2011; van Erp et al., 2012). However, this is not the only reason that may explain such poor performance and reliability. In particular, there is another component of the BCI loop that may also be deficient: the user him/herself who may not be able to produce reliable EEG patterns (Allison and Neuper, 2010). Indeed, it is widely acknowledged that “BCI use is a skill” (Wolpaw et al., 2002), which means the user must be properly trained to be able to successfully use the BCI. Specifically, this is essential for BCI based on the recognition of mental imagery tasks (e.g., motor imagery, Neuper and Pfurtscheller, 2010), the so-called spontaneous BCI, which are the focus of this article^1^. If the user of a spontaneous BCI is indeed unable to correctly perform the desired mental commands, whatever the signal processing algorithms used, there would be no way to properly identify them. Despite this, the BCI community has focused the majority of its research efforts on signal processing and machine learning, mostly neglecting the human in the loop.

In this paper, we argue that the user is one of the most critical component of the BCI loop that may explain the limited reliability of current spontaneous BCI. It does not mean that BCI users are *per se* poor performers or incompetent. It means that the way current spontaneous BCI training protocols are designed is likely to be inappropriate, hindering BCI users to properly learn and use the BCI skill. Indeed, based on a careful analysis of feedback and instructional design literature, we have identified numerous flaws in the design of current spontaneous BCI training approaches. From an instructional design point of view, such flaws are known to impede successful skill learning and may thus explain the poor BCI performances or the fact that some people cannot use a BCI at all [the so-called “BCI illiteracy/inefficiency,” which affects about 20% of BCI users (Allison and Neuper, 2010; Blankertz et al., 2010)].

In this paper, we therefore describe the flaws we have identified in the designs of spontaneous BCI training approaches. Moreover, for each of these flaws, we suggest new research directions that are theoretically expected to address it and, hopefully, to lead to a more efficient learning of the BCI skill. It should be stressed that these suggestions are only based on theory and their related hypotheses. As such, they are not proven solutions, and would require formal validation in the future. Nonetheless, we hope this paper will provoke discussions, debates and more works on this important area of BCI research.

**Figure 1:**
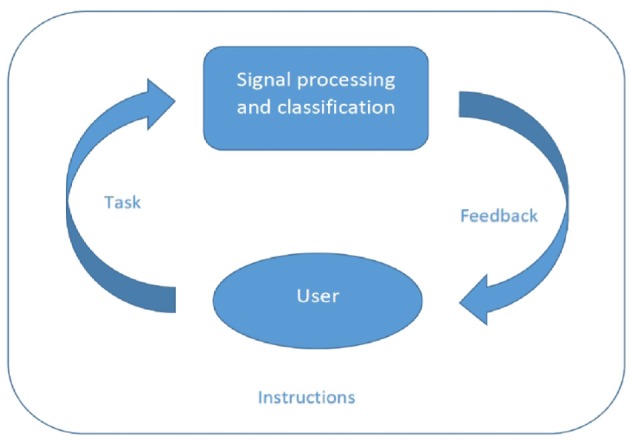
**Conventionally, BCI research is focused mostly on the signal processing and algorithms necessary to translate mental patterns into control commands.** The user and the context in which he or she is learning to produce mental patterns is, on the other hand, often treated with neglect. We argue that the *tasks* a user has to perform, the *feedback* that informs about the performance, and the *instructions* that enable to perform are equally important and discuss them based on literature from instruction design.

This paper is organized as follows: the next section presents a state-of-the-art of human training approaches for spontaneous BCI. Then, the following section identifies the flaws in the design of these classic approaches based on instructional design literature, and suggest new directions to try to overcome them. More precisely, these flaws and suggestions are targeted at different levels of the training approaches (see also Figure 1): at the level of the feedback the user receives, at the level of the instructions provided to him/her, and finally at the level of the training tasks. The last section summarizes the identified flaws and corresponding suggestions and concludes the paper.

## 2. State-of-the-art

Current spontaneous BCI training approaches are rather similar across different BCI designs, and have been mostly the same for years. There have been surprisingly few studies on the impact of various training approaches on BCI performances and user training, in particular as compared to the number of studies on EEG signal processing. Nevertheless, a few interesting research works on feedback and human training approaches have been conducted. This section first presents the common BCI training approaches currently used, then reviews research works that explored alternative approaches.

### 2.1. Current BCI training approaches

BCI control being a skill, it has to be learned, refined and mastered by the BCI user. Neurofeedback^2^ training has been proven to be a necessary component to learn the BCI skill (Neuper and Pfurtscheller, 2010). BCI neurofeedback training principles mostly depend on the type of BCI category used (Wolpaw et al., 2002):

- *The operant conditioning approach*, in which the EEG signal decoder/classifier is fixed and unknown to the user, and this user has to find out how to control a cursor by modulating his/her brain activity in a specific way. Using this kind of approach, the training can last for weeks or even months before the user can control the BCI. This was the approach used to successfully design the first BCI systems (Wolpaw et al., 1991; Birbaumer et al., 1999).
- *The machine learning approach*, in which the EEG decoder/classifier is optimized on examples of EEG signals collected from the user while he/she performs the targeted mental tasks. With this approach the training time before the user can control the BCI is much shorter (about 20 min for 2 classes), see, e.g., (Millán et al., 2002; Blankertz et al., 2006). This is the most used approach.

**Figure 2:**
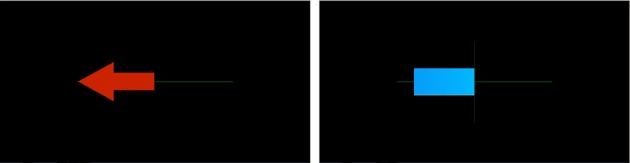
**Example of the display of a classic BCI training protocol. Left**: An arrow pointing left indicates the learner to imagine a left hand movement. **Right**: A feedback bar is provided to the learner. The direction and length of this bar indicate the classifier output and thus the recognized mental task. Indeed, the bar extends toward the left for an identified imagined left hand movement, and toward the right for an identified imagined right hand movement.

These two approaches differ in the way the decoder works (fixed vs optimized on EEG data) and on the instructions provided to the user (e.g., moving the cursor by modulating brain activity in a way to be identified vs performing a given mental task), but the remaining elements of the training approaches are roughly similar. First, the global objective is the same, typically moving an element on screen in different directions depending on the EEG pattern produced. The ways feedback is provided are similar since it is generally a uni-modal (generally visual) feedback indicating the mental task recognized by the decoder together with the confidence in this recognition. It is generally represented by an extending bar or a moving cursor (Neuper and Pfurtscheller, 2010) (see, e.g., Figure 2). Typically, the bar/cursor extends in the required direction if the mental task is correctly recognized and extends in the opposite direction otherwise. The speed of the bar extension or of the cursor movement is also proportional to the decoder confidence in its decision. Finally, the training protocols are also similar. Indeed, with both approaches, the user is trained following a synchronous (or system-paced) protocol, i.e., a protocol in which the user is required to do specific tasks (e.g., extending the bar toward the left by imagining left hand movements) in specific time periods only. The same protocol is usually repeated until the user has learnt the BCI skill, i.e., until he/she has achieved a given performance, usually in terms of rate of correct mental state recognition.

### 2.2. Research on alternative human training protocols for BCI

As we will see later, the training approaches described above have many limitations according to instruction design principles, but are the protocols classically used in current BCI designs. Fortunately, some research groups have explored alternative methods, more in line with instructional design guidelines. We review them below.

Most research on BCI human training approaches so far have focused on studying the impact of various kinds of feedback. In particular, fundamental research on feedback was conducted in the early days of BCI research. Indeed, McFarland et al. showed that feedback was necessary for initial learning of the BCI skill (McFarland et al., 1998). However, they showed that once the BCI skill is learned, then feedback may not be necessary anymore, at least in the short-term and for BCI based on sensori-motor rhythms. They also showed that continuous feedback can have either facilitory or inhibitory effects depending on the learner. The study of Neuper et al. suggested that continuous feedback lead to more efficient BCI learning than delayed discrete feedback (Neuper et al., 1999). In Neuper et al. (2003), they also explored a free training session, in which the BCI user could explore the mental imagery task as he liked, without instructions from the computer. This free training session seemed to have a positive learning impact on the user's EEG patterns (with changes in Event Related Synchronisation/Desynchronisation in the expected direction), although no formal comparison with a training protocol without such session was performed. Kübler et al. used both a continuous feedback during cursor movement and a discrete delayed feedback at the end of each trial, which prove successful to teach BCI users to control their Slow Cortical Potentials (SCP) (Kübler et al., 1999). It is worth mentioning that the discrete delayed feedback was an emotionally charged one, more precisely a smiley face. How an emotionally charged feedback compare to an emotionally neutral one was not formally explored though. Interestingly enough, Kübler et al. also found that, with this kind of training protocol, the performance obtained during early training sessions could predict the number of sessions needed to achieve BCI control (Kübler et al., 2004).

Some authors explored richer and multidimensional feedback, in order to provide BCI users with more information about their brain activity. For instance (Arrouët et al., 2005; Hwang et al., 2009) used as feedback 2D or 3D topography of cortical activation obtained by inverse solutions. Interestingly enough, Hwang et al. have shown that a neurofeedback session in which the user was shown a real-time cortical map of his/her brain activity increased motor imagery-based BCI performances (Hwang et al., 2009). Still exploring multidimensional feedback, Kauffman et al. provided their BCI users with a cursor indicating not only the integrated classifier output, but also its instantaneous sign and absolute value, coded as the color and intensity of this cursor (Kaufmann et al., 2011). Results suggested that users can deal with a multi-dimensional feedback without decrease in performance, although neither without significant increase in performance here. Using BCI with game-like, 3D or Virtual Reality (VR) feedback environments have also been shown to increase BCI performances (Leeb et al., 2006; Lécuyer et al., 2008; Nijholt et al., 2009; Ron-Angevin and Diaz-Estrella, 2009; Lotte et al., 2013). In the same vein, feedback from multiple users playing a BCI-based game together has been shown to increase BCI performances as compared to feedback provided from the user only, during a single-player version of the same game (Bonnet et al., 2013).

Some groups also explored alternative modalities for the feedback, such as tactile feedback (Cincotti et al., 2007) or auditory feedback (Nijboer et al., 2008). Both studies obtained BCI performance similar to that obtained with visual feedback. Some groups also explored multimodal feedback, which combined two modalities. These studies have provided mixed results: a combination of audio and visual feedback has been shown to decrease BCI performances (Hinterberger et al., 2004) while a combination of haptic (a.k.a, proprioceptive) and visual feedback increased performances (Gomez Rodriguez et al., 2011; Ramos-Murguialday et al., 2012).

Some studies showed that biased feedback (i.e., making the user believe he/she did better than what he/she actually did) or positive feedback (i.e., only providing feedback when the task was performed correctly) can improve performances, at least for new or inexperienced BCI users (Kübler et al., 2001; Barbero-Jimenez and Grosse-Wentrup, 2010; Faller et al., 2012). Positive feedback was shown to decrease performance for advanced BCI users though (Barbero-Jimenez and Grosse-Wentrup, 2010), as well as after too many sessions with only positive feedback (Kübler et al., 2001). Vidaurre et al. provided the user with a feedback that was initially generic and progressively more and more specifically tuned for this user (Vidaurre et al., 2011). Indeed, they use a classifier that was initially subject-independent, using a generic set of channels, and progressively adapted the classifier and the channels used to the BCI user. This progressive classifier (and thus feedback) adaptation enabled BCI users initially suffering from the BCI inefficiency to control the BCI.

Aside from work on feedback for BCI, there have been a couple of studies on other components of the BCI training protocol, namely on instructions and training tasks. The work of Neuper showed that specifically instructing the user to perform kinesthetic imagination of movements rather than visual imagination of movements substantially improved performances (Neuper et al., 2005). Concerning training tasks, McFarland et al. successfully used progressive training tasks by first training users to performed 1D control of a cursor, then 2D control and finally 3D control (McFarland et al., 2010).

It should be mentioned that although the training procedure and the signal processing algorithms used are important factors in BCI efficiency, these are not the only ones. In particular, recent works have shown that individual users' characteristics, such as psychosocial and physiological parameters (e.g., gender, instrument playing, fine motor skills) or brain structures, can predict control performances for Mu-rhythm based BCI (Blankertz et al., 2010; Halder et al., 2011, 2013; Hammer et al., 2012; Randolph, 2012).

In summary, although there have been many more research efforts on signal processing and machine learning for BCI, there still have been some interesting research works on training procedures for BCI. As we will see later, several of these work actually comply with guidelines from instructional design literature. Unfortunately, the results from these study are generally not used nor considered in current BCI training protocols. Actually, the BCI training protocols currently used are still the classical ones described in section 2.1, who suffer from many limitations. Indeed they satistify very few of the instructional design guidelines provided by the educational research community, as the next section exposes.

## 3. Flaws in BCI training protocols

Current BCI training approaches, as described in section 2.1, have made BCI control possible, which was a great step forward. Nevertheless, while they made BCI control possible, BCI control still has a poor performance, in terms of speed or accuracy, and many people cannot use a BCI at all (Allison and Neuper, 2010), at least using current training approaches. However, research results in the field of instructional design, educational psychology and human factors have identified the key elements for efficient training across a number of different skills, e.g., language, mathematical, memory or motor skills, making them generic and relatively skill-independent. Even though BCI training approaches are instructional designs (they aim at teaching the BCI skill), most of them unfortunately do not follow guidelines provided by these research fields. As we will see below, they are actually quite far from an ideal instructional design, which may explain the still poor performances of BCI and the high rate of illiteracy/inefficiency. In the following, we analyze the design of BCI training approaches at three levels: (1) at the level of the feedback, (2) at the level of the instructions provided to the user and (3) at the level of the training tasks. For each level, we identify the flaws in BCI approaches according to instructional design literature and propose new directions that are likely to make the designs more efficient.

### 3.1. Feedback

Feedback is known to be a significant factor to motivate learning (Shute, 2008). Moreover, it has been shown that providing extensive feedback to a user leads to efficient and high quality learning (Hattie and Timperley, 2007). However, this is not true for any kind of feedback, and a poorly designed feedback could actually deteriorate motivations and impede a successful learning (Shute, 2008).

What should a good feedback be like then? To be effective, “feedback should be non-evaluative, supportive, timely and specific” (Shute, 2008). It should indicate the user how to improve the task (Shute, 2008) rather than just indicating whether the task was done correctly or not (Hattie and Timperley, 2007; Moreno and Mayer, 2007; Shute, 2008). It should signal a gap between current level of performance and some desired level of performance, hence reducing uncertainty for the user about how he is doing (Hattie and Timperley, 2007; Shute, 2008). In other words, Hattie describes a good feedback as a feedback that can answer the following questions: “where am I going? (what are the goals), how am I going? (progress toward the goal), where to next? (what activities need to be undertaken)” (Hattie and Timperley, 2007). Feedback should also lead to a feeling of competence, in order to increase motivation (whether intrinsic or extrinsic) and thus learning efficiency and efforts (Ryan and Deci, 2000). Finally, an ideal “feedback needs to be clear, purposeful, meaningful” (Hattie and Timperley, 2007).

In contrast, classical BCI feedback satisfies few of such requirements. Indeed, BCI feedback is evaluative and corrective, i.e., it only indicates the user whether he/she performed the task correctly. Also, being only corrective, it does not aim at supporting the user. BCI feedback also does little to help the user feel competent at BCI control. More importantly, BCI is non-specific since it does not explain why or what was good or bad about the task performed by the user. With the machine learning approach, BCI feedback might also be unclear and meaningless, if it is based on a classifier trained on incorrectly performed mental tasks. Unfortunately, this situation is likely, since first time users have by definition never used a BCI before, and thus cannot be expected to perform the required mental tasks perfectly from the start. In other words, for new BCI users who cannot do the mental task correctly from the start, the feedback will indicate them they have done well if they performed the mental task as badly as they did the very first time, during the calibration data collection. It would therefore reinforce bad mental task performance, which is unlikely to be meaningful. Finally, BCI feedback provided during training is often very simple and crude, while during actual BCI operation, to control an actual application, the environment and feedback is often rich and complex. This complexity and environment mismatch may be another source of difficulty for the user.

To work and to be efficient, BCI feedback should therefore be (1) non-evaluative and supportive, (2) meaningful and (3) specific, i.e., explanatory. Additionally, BCI feedback could also benefit from multimodality and more engaging environments.

The need to be non-evaluative and supportive seems to encourage the use of positive feedback, i.e., feedback only provided when the user did well, to let him/her know he/she did well. Hattie indeed recommends the use of positive feedback, at least for beginners and people who want to do the task (as opposed to people who have to do it) (Hattie and Timperley, 2007). For highly self-efficacious learners, Hattie and Timperley (2007) advocates the use of disconfirmatory feedback (a.k.a. negative feedback—i.e., noting when the task was not done properly). The few BCI studies that explored biased or positive feedback obtained results in line with such suggestions. Indeed, they showed that positive feedback was beneficial for new or inexperienced BCI users, but harmful for advanced BCI users (Kübler et al., 2001; Barbero-Jimenez and Grosse-Wentrup, 2010; Faller et al., 2012).

The need to provide meaningful feedback suggests that, in the machine learning approach to BCI, the classifier used should be carefully selected. In particular, if the user initially obtains bad performances, it may be worth not using a classifier trained on the data from this user (which are examples of badly performed mental tasks and thus would lead to feedback reinforcing a wrong strategy). Rather, it could be worth using, at least initially, a subject-independent classifier (Fazli et al., 2009; Lotte et al., 2009), trained on data corresponding to mental tasks correctly performed by other users. In this way, the classifier output is more likely to be a meaningful feedback, indicating (at least roughly) when the user did the mental task correctly. The work on co-adaptive training by Vidaurre et al. is an example of such an approach, with the training protocol starting with generic and subject-independent features and classifier, progressively adapted to the user during training (Vidaurre et al., 2011). It is unclear though whether a subject-independent classifier could be designed for patients, who may have larger inter-subject variability.

More importantly, BCI feedback would theoretically benefit from being specific and explanatory. This means that ideally, the feedback should indicate the user what he/she did well or wrong, and how to improve this. For the moment, BCI feedback is only corrective, which means the user has to figure out what he/she did not do well all by him/herself, without any explanation from the feedback. Since one cannot be easily aware of his own brain activity without neurofeedback, this is likely to be very difficult or even impossible for some users. BCI feedback could therefore provide more information about the brain activity features used by the BCI rather than simply the classifier output (which aggregates everything together). We provide below a couple of suggestions to try to do so:

- Providing as feedback the value of a few relevant features. This would indeed provide a richer feedback, hopefully giving more clues to the user as to what may be going well or not. The number of features shown as feedback should be kept small however. Indeed, an efficient feedback should not be too long nor too complex, and should be provided in manageable pieces (Shute, 2008). Moreover, human working memory being limited to seven information elements at a time on average, one should show less than seven features as feedback (Sweller et al., 1998). Similarly, one could provide the user with a global picture of his/her brain activity, e.g., a 2D or 3D topography of cortical activation obtained by inverse solutions. This has been proved efficient in the study of Hwang et al. (2009).
- Showing users a feedback describing the actual quality of the mental task he/she performed. So far, the quality of the mental tasks has been mostly assessed using classification-based measures, e.g., the distance to the separating hyperplane with linear classifiers. However, this may not be easy to understand for the user. Alternatively, we could identify the properties of a good mental task (e.g., of a good imagined movement), e.g., in terms of strength of the Event Related Desynchronisation/Synchronisation (ERD/ERS) (Pfurtscheller and Neuper, 2001), localization, spatial spread and specificity, stability over time of this ERD/ERS (on this topic, see e.g., Friedrich et al., 2013), etc. Then we would use as feedback a measure of these properties for the task performed by the user. Alternatively, we could also feedback the difference between these properties measures for the current mental task and their value for an optimal mental task. Indeed, such a feedback would actually indicate a gap between current performances (the mental task performed by the user) and a desired level of performance (a good mental task) (Hattie and Timperley, 2007; Shute, 2008). This would also enable to focus on the user's progress, which is recommended (Hattie and Timperley, 2007; Shute, 2008), and thus help him/her to feel competent (Ryan and Deci, 2000).

Current BCI feedback, being mostly visual and unimodal, may also benefit from multimodality. Although research on the benefits of providing learners with multiple representations has produced mixed results, a carefully designed multimodal feedback may prove useful (Ainsworth, 2006; Merrill, 2007). As mentioned in section 2.2, research on multimodal feedback for BCI has also produced mixed results. These mixed results are well summarized by Ainsworth, who mentioned that “By switching between representations learners can compensate for weaknesses in their strategy. However, if learners are attempting to relate different representations, then this may provide a source of difficulty” (Ainsworth, 2006). This work also suggests that the content of the representations may be more important than the modalities used for each representation (Ainsworth, 2006). In particular, an efficient multimodal representation should use the same formats and operators on each representation, i.e., one should be able to interpret the different representations in a similar way, using the same kind of mental analysis (Ainsworth, 2006). The different representations should also have a similar specificity, i.e., the same granularity of explanatory content (Ainsworth, 2006). Finally, there should be some redundancy between representations so that the user can easily relate them (Ainsworth, 2006). This suggests that a multimodal BCI feedback respecting these guidelines might be useful. For instance, the work in Hinterberger et al. (2004) used different granularity for the auditory and visual modalities, the visual feedback being continuous while the audio one was discrete. This might explain why it decreased BCI performances. On the contrary, the works in Gomez Rodriguez et al. (2011) and Ramos-Murguialday et al. (2012) used the same granularity for both visual and haptic feedbacks, which increased BCI performances.

It should also be mentioned that high quality learning also requires authentic motivation (Ryan and Deci, 2000). This means the feedback and the feedback environment should be inherently motivating and relevant for the learner and have an appeal of novelty, challenge, real-world relevance or aesthetic value (Ryan and Deci, 2000; Merrill, 2007). This supports the use of more engaging feedback environments rather than boring and basic feedbacks such as a classic bar or cursor feedback. Results observing that using BCI with game-like, 3D (even in non-immersive settings) or Virtual Reality (VR) feedback environments increase performances are thus in line with these recommendations (Lécuyer et al., 2008; Lotte et al., 2013). This may also be expected to help the user getting used to richer and more complex environments, thus lowering the mismatch between the feedback provided during training and during real-world use.

### 3.2. Instructions

According to instructional design, BCI training approaches could also be improved at the level of the instructions provided to the user before actually starting the training. Indeed, in current BCI training procedures, instructions are rarely considered, and often not mentioned in the papers. Most of the time they consist in asking the subject to perform the targeted mental tasks, or to move the cursor or bar in the required direction. An important exception is the work of Neuper et al. on the necessity to instruct users to perform kinesthetic rather than visual motor imagery (Neuper et al., 2005). This suggests that instructions are important, which is confirmed by instructional design literature (Hattie and Timperley, 2007; Shute, 2008). Indeed, it is known that feedback is more effective when goals are clearly defined and specific (Hattie and Timperley, 2007; Shute, 2008). This stresses that when providing instructions about the BCI training procedure to a user, we should also clearly state the goals and objectives of the training. The objective of a BCI training session may not really be to move a bar left or right nor to imagine movements. Rather, it should be to help the user in producing clear, specific and stable brain patterns. This goal could therefore be explicitly mentioned to the user so that he/she knows the targeted direction and thus what is expected from him/her. In this way he/she would benefit more from the feedback to reach this goal.

Instructional design literature also stresses the need for pretraining or at least initial knowledge or experience on which the training can be based and built (Hattie and Timperley, 2007; Merrill, 2007; Moreno and Mayer, 2007). In the same vein, it is also recommended to demonstrate the knowledge or skill to the student before he actually learns to master it (Merrill, 2007). Both this initial experience and demonstration are usually missing in BCI training protocols. This suggests that BCI training might be made more efficient by, e.g., before the actual BCI practice, instructing the subjects to remember a situation in which they may have used the task they will mentally imagine to drive the BCI. For instance, in the case of motor imagery-based BCI, at the beginning of a session subjects could be instructed to vividly remember a situation in which they performed a given movement (e.g., during a sport session) before imagining it during the subsequent BCI use. This would activate their prior experience with the task they will imagine, which is expected to make the learning easier (Merrill, 2007). Interestingly enough, Halder et al. showed that the ability to recall sensorimotor programs was indeed correlated to BCI performances (Halder et al., 2011). Similarly, showing the BCI learner a demonstration of a successful BCI use, together with a demonstration of BCI feedback during correctly performed mental tasks (see section 3.1), might also promote the learning of the BCI skill (Merrill, 2007).

Feedback itself is also an element on which instructions could be provided. Indeed, for the feedback to be efficient, the learner should understand the representations involved (Ainsworth, 2006). For the learner, this can involve learning to ignore potentially erroneous intuitions that he/she may have about the meaning of the feedback. Some researchers even argue that learners should be taught how to interpret and understand the representations and thus the feedback (Ainsworth, 2006). This suggests that instructions should also be provided to the BCI users in order to explain them the meaning of the feedback. This seems particularly important if the feedback is related to a classifier output, whose actual meaning (e.g., the distance to a separating hyperplane) is unlikely to be intuitive for people not familiar with classification, i.e., for most real-life BCI users.

### 3.3. Tasks

The last part of BCI instructional design that could be improved is related to the tasks users have to complete. As mentioned before, BCI training tasks are mostly synchronous (a.k.a., system paced) and repeated identically until the users has learned the BCI skill. However, research on education and learning recommends to follow a different approach (Sweller et al., 1998; Ryan and Deci, 2000; Ainsworth, 2006; Hattie and Timperley, 2007; Shute, 2008).

In their book “The media equation,” Reeves and Nass (1996) showed that we respond similarly to mediated reality and to real world equivalents: As boring and repetitive teachers are seldom inspiring the engagement and attention necessary for an optimal learning experience, boring and repetitive learning programs have the same effect. Accordingly, to increase the efficiency of computer mediated learning, and specifically of BCI-control learning, the user needs to be presented with an involving and engaging learning environment.

Accordingly, Nijboer et al. (2008) have shown that mood and motivational factors, such as mastery confidence and incompetence fear, are relevant for learning to control a SensoriMotor Rhythm (SMR) BCI. In a longitudinal patient study, Nijboer et al. (2010) found that an increase of SMR-BCI performance correlates with the motivational factor of challenge. Similarly, Kleih et al. (2011) found that motivational factors of challenge and incompetence fear correlate positively with SMR-BCI performance. During learning tasks, different types of motivation can increase the engagement and efficiency of the user (Ryan and Deci, 2000). The strongest motivation, intrinsic motivation, is anchored in the individuals most basic urges: the feeling of competence, autonomy, and relatedness. By appealing to these basic needs in the task construction for BCI, the user's motivation and task engagement can be increased.

To increase the feeling of competence, in general, training tasks should be progressive and adaptive: the learners should first manipulate the least complex representations and should then be progressively introduced to new representation as his/her expertise grows (Ainsworth, 2006; Merrill, 2007). In a similar fashion, the training protocol should provide the user with assignments that are challenging (Hattie and Timperley, 2007), but still achievable (Shute, 2008). Finally, studies have revealed that students could increase their efforts if these can lead to more challenging tasks or higher quality experiences (Hattie and Timperley, 2007). This supports that BCI training protocols and tasks could benefit from being adaptive, with a difficulty that increases as the user increases his/her skills with BCI. For instance, the user could be asked to try out a single mental task at the beginning, rather than all of them at once. Then, he/she will be asked to perform different mental tasks as he/she starts to master the initial ones. The adaptive training protocol of McFarland et al. (1D-control, then 2D, then 3D) made 3D cursor control possible with EEG-based BCI for the first time (McFarland et al., 2010), which seems to support the need for progressive training tasks. Moreover, it has been shown that scaffolding also enhances learning in early stage of training, but should be removed in later stages (Shute, 2008). For instance, in cases where a motor imagery-based BCI is used by individuals with residual motor capabilities or by non-handicapped users, real movements can be used as a scaffold for motor imagery (Higashi et al., 2011). It is an easy-to-manage starting point for training, which then can be slowly replaced by quasi-movements using an EMG-biofeedback approach (Nikulin et al., 2008). Such a gradual transfer from well-known or simple tasks to new tasks initially minimizes the cognitive demand on the users during training, and hence the risk to frustrate and demotivate them.

Regarding the feeling of autonomy, several authors stressed that offering learners the possibility to proceed at their own pace increases their motivation and makes them learn more efficiently (Ryan and Deci, 2000; Moreno and Mayer, 2007; Shute, 2008). This suggests that BCI training protocols could include more free or even self-paced BCI sessions. In other words, users could benefit from being offered—at least from time to time—the possibility to decide the mental task they will perform, rather than always doing the one instructed by the program. They could be offered to do so either when instructed by the computer (i.e., using a so-called synchronous BCI) or, which should be even better, whenever they want too (i.e., using a so-called asynchronous/self-paced BCI). Moreover, self-paced BCI sessions would give time to the users to reflect upon the mental task they did and the corresponding feedback received, which is also recommended for efficient learning (Moreno and Mayer, 2007). Neuper et al. explored such a free self-paced session with a single patient and obtained positive results (Neuper et al., 2003). Although no formal comparison with classical approaches were performed in this study, this would still suggest that including self-paced sessions may prove useful for BCI training.

Related to the mood and motivation of the user is the creation of an emotionally appealing task environments during the learning process. Um et al. (2012) showed that these can facilitate learning by the creation of positive emotions. Theoretically, the impact of emotions on learning can be divided into quantitative effects, e.g., on long-term memory retention, and qualitative effects, e.g., on cognitive organization and creativity. For BCI task acquisition, the retention of the performed mental task and a flexibility during the learning process, e.g., trying different variations of the mental tasks at hand, might be relevant factors that lead to increased performance of the subject. Additionally, excitement and interest created by appealing task environments, such as computer games can also increase the level of activation and engagement of the user (Plass-Oude Bos et al., 2010). However, it is not necessarily the case that negative emotions have a negative impact on learning. Kort et al. (2001) mention that negative emotions or cognitive-emotive states can be useful and integral parts of the learning process. For example, they can lead to an activation of the learner, and initiate changes in an unsuccessful approach or the “unlearning” of false and impeding beliefs. On the other hand, there is also evidence for the detrimental effects additional emotional information can have on the learning process. The reason for these negative effects of emotion during learning is assumed to lie in the additional load on working memory that emotional information can pose and on the interference with the main learning task. Care should therefore be taken when adding emotion-inducing elements to the learning task (Um et al., 2012).

Furthermore, educational research has shown that variability over training tasks and problems encourages the learners to build abstractions since it increases the probability to identify useful features and strategies and to distinguish them from irrelevant ones (Sweller et al., 1998; Ainsworth, 2006). This suggests that BCI training tasks could also include variety in the tasks the users have to complete. Rather than doing exactly the same tasks over and over again, e.g., imagining the same left and right hand movements, the users could be asked to perform slightly different tasks from one trial to the next. For instance, the user would still be asked to perform imagined movements, but he/she could be asked to vary the speed of the imagined movement, its strength, the duration of the imagination, the gesture imagined, etc. This may help the user identifies successful mental strategies as well as the important characteristics of a good mental task.

Finally, it is also known that every student is different and thus that ideally, different training procedures should be used for different people (Hattie and Timperley, 2007; Merrill, 2007; Shute, 2008). As such, among the different variations of training protocols mentioned, it could be necessary to identify—through experiments—those that are the most appropriate for which kinds of users' characteristics. These characteristics describe important features of the learner, either cognitive, psychological or physiological, that might influence the way they use and learn BCI, such as age, gender, education level, video game experience, spatial abilities, etc. [see, e.g., Larrue et al. (2012) where users' characteristics where controlled in a study comparing navigation in VR with a BCI and with a treadmill]. A few studies have found correlations between psychological parameters and SMR-BCI control performances (Hammer et al., 2012; Randolph, 2012), which would suggest that matching users' characteristics to the corresponding BCI type is likely to optimize control performances. Similarly, matching training protocols to users' characteristics may make BCI training more efficient.

On a more prospective side, it has been observed that people regularly exposed to video games had improved visual and spatial attention, memory and mental rotation abilities (Green and Bavelier, 2003; Feng et al., 2007; Boot et al., 2008). Extensive video-game practice has also been shown to improve the efficiency of movement control brain networks and visuomotor skills (Granek et al., 2010). Since these various skills are involved in some mental tasks used to drive BCI [e.g., mental rotation of geometric figures, motor imagery, remembering familiar faces, … (Lotte, 2012; Friedrich et al., 2013)], this suggests that BCI users might improve their mastery of BCI by performing training tasks that do not involve the BCI system, such as by playing various video games. To the best of our knowledge, correlation between regular video game practice and BCI performance has not been shown yet for BCI based on mental tasks, but has been observed for BCI based on Steady-State Visual Evoked Potentials (SSVEP) (Allison et al., 2008). This suggests that having BCI users practicing (non-BCI-based) video games might be a promising training task to improve their BCI control skills.

## 4. Conclusion

Based on a study of educational psychology and instructional design research papers, we have highlighted that BCI training approaches were very likely to be inappropriate and may benefit from multiple improvements that could increase BCI performances and reduce BCI illiteracy/inefficiency. We have identified the flaws of BCI training protocols from the perspective of instructional design and proposed some suggestions that are theoretically expected to address these flaws and make BCI training more efficient. Naturally, these suggestions are only based on instructional design principles and would need to be formally explored and validated to assess their actual efficiency. The properties of a good training protocol and the corresponding suggestions for BCI training are summarized in Table 1. Overall, we suggest to provide a BCI feedback that is (1) positive feedback in early training stage and disconfirmatory in later stages, (2) meaningful, i.e., not related to the output of a classifier trained on incorrectly performed mental tasks, and (3) specific and explanatory, i.e., which provides the user more information about his/her brain activity than the classifier output. Instructions may be improved as well, by defining a clear and specific learning objective and explaining it to this user. Instructions may also be provided to explain the feedback meaning, to instruct the subject to activate prior experience with the task he/she will use, and to demonstrate correct BCI use. Finally, BCI training tasks may also be improved by (1) being adaptive with increasing complexity and difficulty, (2) including self-paced sessions, (3) being more engaging and (positive) emotion-inducing, (4) including a variety of tasks, and (5) matching users' characteristics. We also showed that the few papers that studied BCI training procedures are generally in line with these recommendations derived from instructional design literature. This further stresses the relevance of working on BCI instructional design. In turn, this also suggests that training protocols for BCI studies and designs should deserve more attention. As such, we would recommend BCI authors to carefully describe the training protocols they use in their papers, so that the whole BCI design could be fairly understood and assessed. Similarly, BCI training protocols, as many BCI components, would benefit from standards, so as to enable fair comparisons between BCI designs.

**Table 1:** **Summary of desirable properties of a good instructional design with corresponding suggestions to improve human training protocols for BCI**.

**Level**	**Properties of a good instructional design**	**Corresponding suggestions for BCI training protocols**
Feedback	- Non-evaluative and supportive feedback (Hattie and Timperley, 2007; Shute, 2008)	Provide positive feedback (feedback only indicating when the user did right) only for beginners, and disconfirmatory feedback for advanced users
	- Feedback that conducts to a feeling of competence (Ryan and Deci, 2000)	
	- Clear and meaningful feedback (Hattie and Timperley, 2007)	Start with a subject-independent classifier for users with poor initial performances
	- Explanatory and specific feedback (Hattie and Timperley, 2007; Shute, 2008) (Moreno and Mayer, 2007)	Provide more information about what was right or wrong about the EEG patterns produced by the user:
	- Feedback that signals a gap between current and desired performances (Hattie and Timperley, 2007; Shute, 2008)	- Provide as feedback the value of a few (less than seven) relevant EEG features
		- Provide as feedback some measure of quality of the mental imagery
	- Multimodal feedback (Ainsworth, 2006) (Merrill, 2007)	Provide a multimodal feedback (e.g., visual + haptic), with the same granularity and specificity for each modality, with some redundancy between them
	- Engaging feedback and environment (Ryan and Deci, 2000)	Represent the feedback as an interaction with a game element (e.g., a 3D car)
Instructions	- Goals should be clearly defined (Hattie and Timperley, 2007; Shute, 2008)	Expose the real goal of BCI training, i.e., to produce clear, specific and stable EEG patterns
	- The meaning of the feedback should be explained (Ainsworth, 2006)	Explain what the BCI feedback means, particularly for non-intuitive feedback such as the classifier output.
	- Prior knowledge should be activated (Merrill, 2007; Moreno and Mayer, 2007)	- Instruct the users to remember situations in which they used the task they will imagine
	- The skill to be learned should be demonstrated (Merrill, 2007)	- Demonstrate successful BCI use and BCI feedback during correct task performance
Tasks	- Progressive and adaptative tasks (Ainsworth, 2006; Merrill, 2007)	Use adaptive BCI training protocols with increasing difficulty (e.g., progressively increasing the number of mental tasks to be mastered)
	- Tasks that are challenging but still achievable (Hattie and Timperley, 2007; Shute, 2008)	
	- Need for autonomy and work at the user's own pace (Ryan and Deci, 2000; Shute, 2008) (Moreno and Mayer, 2007)	Include more training sessions with free and/or self-paced BCI use
	- Motivation and positive emotions promote learning (Ryan and Deci, 2000; Um et al., 2012)	Using positive emotion-inducing training tasks e.g., including gaming mechanisms
	- Need for variability over tasks and problems (Sweller et al., 1998; Ainsworth, 2006)	Include variety in the mental tasks to be performed, e.g., change in speed or duration of the mental imagery
	- Adapt the training procedure to the student (Hattie and Timperley, 2007; Shute, 2008)	Matching BCI training protocols to users' characteristics

It should be noted that such suggestions are only based on theory, and will need to be formally validated.

With this literature study, we hope to provide a new perspective on the well-known performance issue of BCI. We also hope that this will bring the BCI community attention to a mostly neglected aspect: much still needs to be explored about training procedures for BCI, which also means that BCI performances still have much potential for further improvement. We provide here a number of suggestions for further research, which we expect will contribute to motivate researchers to explore these areas and to further advance the field of BCI design.

### Conflict of interest statement

The authors declare that the research was conducted in the absence of any commercial or financial relationships that could be construed as a potential conflict of interest.

## Footnotes

^1^In this article, we do not consider BCI based on Event Related Potentials, such as P300-based BCI. Indeed, these latter rely on brain responses evoked by external stimulus and as such they involve practically no human training (Wolpaw et al., 2002; Fazel-Rezai et al., 2012).

^2^Neurofeedback consists in providing the user with a real-time feedback about his/her own brain activity so that he/she can learn to voluntarily control it.
